# Addressing mental health issues among migrant and refugee pregnant women: A call for action

**DOI:** 10.18332/ejm/108626

**Published:** 2019-05-02

**Authors:** Maria Iliadou, Maria Papadakaki, Eirini Sioti, Paraskevi Giaxi, Evangelia Leontitsi, Elena Petelos, Maria Van den Muijsenbergh, Styliani Tziaferi, Anastasios Mastroyiannakis, Victoria G. Vivilaki

**Affiliations:** 1 Department of Midwifery, Faculty of Health and Care Sciences, University of West Attica, Athens, Greece; 2 Department of Nursing, Laboratory of Integrated Health Care, Faculty of Human Movement and Quality of Life Sciences, University of Peloponnese, Sparti, Greece; 3 Department of Social Work, School of Health and Social Welfare, Technological Educational Institute of Crete, Heraklion, Greece; 4 Clinic of Social and Family Medicine, Department of Social Medicine, School of Medicine, University of Crete, Heraklion, Greece; 5 Department of Primary and Community Care, Radboud University Nijmegen Medical Centre, Nijmegen, The Netherlands; 6 CMT–Prooptiki, Athens, Greece

**Keywords:** mental disorders, pregnant, well-being, healthcare professionals, refugee, migrant

## Abstract

Migrant and refugee pregnant women constitute a highly vulnerable group to mental disorders. The rates of mental illness of migrants and refugees are higher than those of host populations, with migrant women being more likely to suffer from prenatal depression. A Policy Paper was developed based on a literature review conducted in Medline, Scopus and Google Scholar. Filtering criteria were: year of publication (2002–2017), study topic relevance, and English language. A total of 63 documents were identified. Most of the documents were scientific papers while a large number of documents were reports of EU committees and networks on migrant issues or annual reports of international bodies. From the analysis of existing evidence, four major topics emerged for the perinatal health of migrant women: *1) Prevalence and risk factors for antenatal mental disorders, 2) Assessment of mental disorders, 3) Healthcare professionals’ training on supporting migrant and refugee pregnant women, and 4) Interventions for the mental health of migrant women. Midwives and other members of interdisciplinary teams have to be trained and culturally competent to successfully meet the needs of migrant and refugee pregnant women.*

## COMMENTARY

The number of international migrants worldwide reached 244 million in 2015, and the total number of refugees was estimated at 19.5 million in 2014^1^. These population groups face higher rates of physical and mental illness than the respective host populations^2^. Female migrants comprise 48 per cent of all international migrants^1^, with migrant and refugee pregnant women forming a highly vulnerable group to mental disorders, due to their unique needs during this period^3^.

Although perinatal mental health is recognized as a significant public health issue^4^, its complexity and diversity make it difficult to understand or manage^3^. Women have an increased risk of mental illness during the perinatal period, with implications to themselves, their infants, families, and society. Especially during the antenatal period, mental disorders constitute a common complication of pregnancy^5^.

Only by understanding how migration is related to mental illness can we establish how it might be detected and treated and what interventions should be developed and implemented to combat it successfully. With the above in mind, the purpose of this synthesis is to summarise the available data concerning mental health status among migrant and refugee pregnant women and the role of healthcare professionals in addressing this issue.

A literature review was conducted in Medline, Scopus and Google Scholar, using the combinations of the following mesh terms: ‘mental health’, ‘perinatal’, ‘immigrant/migrant pregnant’, ‘refugee pregnant’, ‘healthcare professionals’, and ‘interventions’. The search was conducted in the keywords, abstracts and titles of articles, between July and August 2017. Inclusion criteria included: the year of publication (2002–2017), study topic relevance, and English language. In addition to the above, snowball searches were performed in the websites of national and international professional colleges and associations as well as institutes and organizations in the field of health [e.g. National Institute of Health and Care Excellence (NICE); American College of Obstetricians and Gynecologists (ACOG); Canadian Medical Association (CMA); Royal College of Obstetricians and Gynecologists (RCOG); World Health Organization (WHO)]. Two reviewers initially screened the titles and abstracts of all the articles for relevance to the review topic. The articles that underwent the screening phase were read in full and analysed for the key message. The documents located through the electronic search were organised based on the type of document they represented. The analysis followed the principles of content analysis.

**Figure 1:**
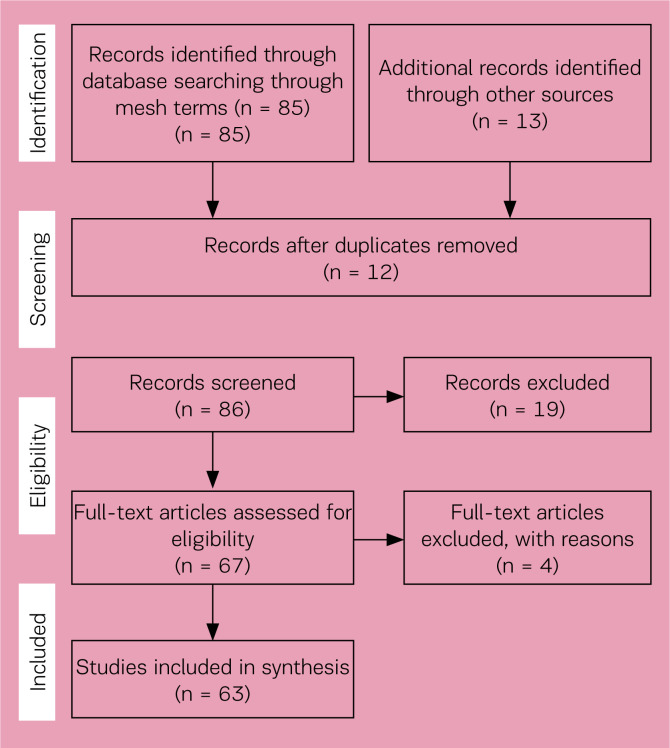
Prisma flow diagram

Even though this review is not a systematic review, a flow diagram of the search selection for the included studies according to the Preferred Reporting Items for Systematic Reviews and Meta-Analysis (PRISMA) statement^6^ is presented in Figure 1. A total of 63 documents were identified and met the inclusion criteria, focusing on the perinatal health of migrant, refugee and asylum-seeking women. Documents included scientific papers and grey literature including reports of EU committees and networks on migrant issues or annual reports of international bodies.

From the analysis of existing evidence, four major topics emerged for the perinatal health of migrant women: 1. Prevalence and risk factors for antenatal mental disorders, 2. Assessment of mental disorders, 3. Healthcare professionals’ training on supporting migrant and refugee pregnant women, and 4. Interventions for the mental health of migrant women.

### 1. Prevalence and risk factors for antenatal mental disorders

Mental disorders are encountered more frequently in women than in men^7^. Common perinatal mental disorders have a higher prevalence in low- and middle-income countries, particularly among poorer women with genderbased risks or a psychiatric history^8^. The percentage of women that experience a mental disorder is about 10% worldwide^8^. Although current evidence suggests that the rates for depression have no differences in perinatal and non-perinatal populations, the rates for anxiety disorders and bipolar disorder may be slightly higher in the perinatal population^9^.

Depression is one of the most common complications during pregnancy, with a higher incidence of major depressive disorder episodes (MDD) and depression onset during the childbearing years^10,11^. The prevalence of MDD, specified by diagnostic criteria during pregnancy is 12.7%, while 37% of women report experiencing depressive symptoms while being pregnant. The prevalence of depression is 8.5–12.9% in high-income countries^12-15^, though in low- and middleincome countries rates are increased to 15.6–25.8%^15-17^. The most significant predictors for antenatal depression are low self-esteem, antenatal anxiety, low social support, negative cognitive style, major life events, low income and history of abuse^18^. Although anxiety is known to have a higher prevalence than depression at all stages of pregnancy, there is a high level of comorbidity between them at approximately 60%^10,11,19^. When a mother experiences depression, anxiety, or stress during pregnancy, she may expose both herself and her infant to multiple psychological risks^11,20^, including impaired bonding with the fetus and the newborn, increased risk of poor psychological postnatal adjustment, postnatal depression^11,21^, and physiological consequences, including low birth weight^22,23^, intrauterine growth restriction, preterm birth^22-26^ and also consequences that involve family relations and wider society^27^.

As far as it concerns socioeconomic determinants about mental health for these adverse outcomes, the correlation becomes even stronger when it comes to low- and middle-income countries (LMICs) and generally among lower socioeconomic groups^28^. According to evidence, the rates of mental illness of migrants and refugees are higher than those of host populations^3^,^4^,^29^ with one in three migrant women being affected by depression15 and being more likely to suffer from prenatal depression^30^.

As migrant women are more likely to be poorer financially, and most global migration is from LMICs, we may expect worse adverse child outcomes associated with perinatal mental disorders among migrant women^31^. Besides, being a pregnant migrant woman can be a source of cultural and psychological distress, which can, further, lead to the immediate or delayed manifestation of mental health disorders, either in them or their offspring^32^.

Risk factors for depression in migrant pregnant women identified in the recent systematic review by Anderson et al.^31^ are lack of social support, marital strain/lack of marital support, time in host country, socioeconomic difficulty (including lack of money for basic needs and housing difficulties), stress/mental health, low acculturation level, not working or attending school in pregnancy and precarious legal status.

Due to cultural and linguistic barriers, migrants may be unwilling or feel unable to seek help, and those who are eager to do so are often not aware of the services available to them. These women present commonly with somatic symptoms and are left in social isolation. In general, they tend to prefer practical help instead of pharmacological interventions^33^.

Migrant pregnant women who experience single-parenthood or psychological difficulties, which may be associated with social or psychological vulnerability, require special attention, as they are disproportionately likely to use psychoactive substances like alcohol and tobacco, and these put them and their children at high risk of poor health outcomes^34^. Migrant pregnant women with a low acculturation level are less often smokers and women with a high level are more often smokers than native women^35^. In studies from the United Kingdom, Sweden, the United States, and Turkey, it was found that smoking increases with increasing acculturation^35-38^. Prevention measures have to prevent women with a low acculturation level from starting to smoke and induce those with a high acculturation level to quit. Smoking and acculturation are group phenomena. Smoking, in interpersonal level, is influenced by peer group social norms, by the tobacco control policies in each country and by the social support systems. Also, acculturation is a dynamic process depending on interactions between immigrants and natives^39^. Hence, it is vital to engage migrant communities in the prevention process^35^. The qualitative study of Fellmeth et al.^29^ showed that mental illness was recognised as a concept by the majority of participants, who were migrant and refugee pregnant women. Moreover, mental illness was thought to be more prevalent during and following pregnancy owing to lack of family support and concerns about the future^29^.

Moreover, when it comes to asylum seekers, the risk of violence during pregnancy is associated with a higher risk of prenatal depressive symptoms^40^ and it is less likely to be reported^41^. A first step in preventing mental health problems is to recognise violence as a clinically relevant and identifiable risk factor for antenatal depression, significant or minor, in migrant women. Healthcare providers who care for pregnant women can be trained to identify and to appropriately direct women and their families to the available services^30^.

Also, the stress associated with the legal processes around asylum-seeking procedures and the fear of an unsuccessful claim and subsequent deportation can further damage the pregnant woman’s mental and physical health^42^. The asylum-seeking procedure itself and the accompanying poverty and deprivation together with the possible emotional impact and cultural ramifications, if, for example, the woman is pregnant as a result of rape, can lead to these women experiencing psychological issues including depression, anxiety and post-traumatic stress disorder^43^.

Establishing the prevalence of depression in pregnant and post-partum migrants and refugees is essential for the development of appropriate mental health services^29^. Although there is an increasingly hostile environment towards migrants, society must be involved to confront discrimination, poverty and social isolation that migrant women suffer^31^.

### 2. Assessment of mental disorders

The establishment of routine screening for abuse in the maternity services settings, as well as tested, proper, culturally sensitive referral systems are needed^30^. Clinicians need to be aware of psychosocial issues in this vulnerable population and be able to conduct careful screening and follow-up^33^. According to NICE^44^, midwives should provide information when booking the appointment for women with a previous severe mental health problem (severe and incapacitating depression, psychosis, schizophrenia, bipolar disorder, schizoaffective disorder or postpartum psychosis) or any current mental health problem; information should be about how their mental health problem and its treatment might affect them or their baby. Women who cannot communicate in English should be provided with equal access to information with the help of interpreters, supported by cultural mediators if possible, or other appropriate local support available, as provided by independent advocates in the United Kingdom, when needed.

Also, general practitioners (GPs), midwives, health visitors and obstetricians should ask all women about their emotional state at each routine antenatal contact to support identification and discussion of mental health problems. In light of the above, possible identification questions (Table 1) that could be asked by healthcare professionals when booking the woman’s appointment and routine visits in pregnancy, focusing on her mental health and well-being have been recommended by NICE^45^.

**Table 1:** Depression identification questions

During the past month, have you often been bothered by feeling down, depressed or hopeless?
During the past month, have you often been bothered by having little interest or pleasure in doing things?
**Questions on anxiety using the 2-item Generalized Anxiety Disorder scale (GAD-2)**
Over the last 2 weeks, have you been feeling nervous, anxious or on edge?
Over the last 2 weeks, have you not been able to stop or control worrying?

NICE. Antenatal and postnatal mental health: clinical management and service guidance. Clinical guideline [CG192]. London: National Institute of Health and Care Excellence, 2014.

Routine depression screening at antenatal care visits (in each trimester of pregnancy)^46,47^, as well as information on immigration indicators (duration of residence in the host country, language fluency, legal status as a proxy indicator of socioeconomic condition and difficulties, religion and ethnicity) and psychosocial risk factors (living conditions, social and marital support), are crucial and should be considered an essential part of routine perinatal health data collection^48,49^, especially when providing care to refugee women^49^. It is important that women are interviewed privately, to avoid the possibility of self-censorship. This requires the availability of cultural mediators as well as competent interpreters and healthcare professionals^50,51^. Moreover, in primary care settings, pregnant migrant women should be provided with descriptive materials, in their mother tongue, regarding depressive disorder, receive translated screening questions and have access to trained interpreters, ideally supported by cultural mediators, to facilitate the diagnostic interview, and systematic inquiries about losses, stressors and symptoms^42-51^.

Also, GPs and mental health professionals should carry out comprehensive mental health assessments for women who may have a mental health problem in pregnancy to aid diagnosis and identify the need for extra support. Especially for women with diverse cultural backgrounds, NICE^45^ recommends that *‘healthcare professionals should be culturally competent in their discussions with them in order to support full discussion and comprehensive mental health assessment and women should have access to an interpreter, supported, if possible, by a cultural mediator or […] independent advocate as in the case of UK, if needed’*. Furthermore, when a woman is referred to mental health professionals, she should be assessed within two weeks of referral and start treatment within six weeks of referral. These assessments and interventions should be culturally designed to be understood and communicated effectively.

### 3. Healthcare professionals’ training in supporting migrant and refugee pregnant women

With regard to mental healthcare provision in migrant and refugee pregnant women, there are various barriers to healthcare systems, as free care can be extremely limited in some cases. However, this specific review mainly focuses on the healthcare professional’s important role and needs^52^.

Asylum seeking pregnant women may not always have positive experiences of maternity care, and midwives and other professionals may not be able to meet their special needs^53-56^. NICE^45^ suggests that healthcare professionals should be given training on: the specific health needs of women who are recent migrants, asylum seekers or refugees; the specific social, religious and psychological needs of women in these groups; the most recent government policies on access and entitlement to care for them. Also, healthcare professionals should offer the woman information on access and entitlement to healthcare, discuss with the woman the importance of having her hardcopy maternity record with her at all times at the booking appointment, and avoid making assumptions based on the woman’s culture, ethnic origin or religious beliefs. Besides, healthcare professionals should provide the woman, who does not speak or read English, with access to an interpreter (who may be a link worker or advocate as in the case of UK and should not be a member of the woman’s family, her legal guardian or her partner)^44^. Moreover, health care professionals should communicate with the woman in her preferred language, and when giving spoken information, they should ask the woman about her understanding of what she has been told to ensure she has understood it correctly^44^. It is crucial to improve the training of perinatal healthcare professionals to encourage the development of maternity-linked transcultural sensitivity and competence. Additionally, recent work conducted in a capacity-building project focusing on refugee health (EUropean Refugees-HUman Movement and Advisory, EUR-HUMAN Project, http://eur-human.uoc.gr) reports that compassion is a critical competency for healthcare professionals treating refugees and migrants, and highlights the fact that linguistic and cultural barriers exacerbate the lack of compassion in care, especially where healthcare information and psychological support are necessary and where appropriate supporting frameworks are missing.

Moreover, as the next generation of midwives, pre-registration students also require adequate preparation in order to adequately care for asylum-seeking women^57^. *‘The pregnant woman within the global context’* model for midwifery education can be used in midwifery education to prepare students in caring for pregnant women seeking asylum. This model was designed to facilitate midwives to visualise the woman as the centre of her care. It incorporates broader factors, on a global level, which could impact on the health and social care needs of a pregnant woman in the process of seeking asylum. It also prompts students to consider the influence of existing narratives and dominant discourses on perceptions of asylum-seeking and is designed to encourage students to question such discourses. It has been designed as a tool to assist midwifery students in assessing the health and social-care needs of pregnant women seeking asylum^57^.

### 4. Interventions for the mental health of migrant women

Among socioeconomically disadvantaged populations, the need for early identification of pregnant women with mental disorders and provision of early interventions and prevention research is highly punctuated^58^. Improving communication and allowing migrant women to preserve some of their traditions may increase their mental wellbeing^59^. Peer support groups reduce social isolation and feelings of desperation due to immigration and family separation. This could be an effective way of preventing postpartum depression among migrant women^60^. Moreover, the availability of childcare facilities, transportation and support from family members and spouses can facilitate seeking help. Group meetings can be an effective way to provide social support and health-promotion information^47^. Besides, mind-body interventions might alleviate women’s anxiety during pregnancy^61^ and physical exercise may be an effective way to treat depression symptoms during pregnancy^62^. Guidance from NICE^4^ states that health professionals should consider exercising treatment for antenatal depression, and the RCOG^63,64^ and the ACOG^65^ have stated that exercise can provide mental health benefits during pregnancy. Also, group programs that incorporate education about mental and physical health, available support, and socialization, appear to be efficient in engaging and assisting migrant perinatal women. According to evidence, these are best delivered by clinicians from similar cultural backgrounds^33^. It is further suggested that more formal and systematic training, including the development of assessment tools in the local languages, would enable better identification and treatment of mental illness in this population^29^.

Moreover, a feasible way of overcoming the treatment barriers of perinatal mental health disorders and making sure that women obtain the care they need is by integrating perinatal mental health services into primary care. Perinatal mental health is central to the values and principles of Midwifery, and the establishment of community midwifery services for perinatal mental health is inexpensive and quite beneficial. Community midwives can effectively assess, recognise, support and refer women with perinatal mental disorders and thus provide holistic women care^66^.

This discussion piece is not intended to be exhaustive and developed as a systematic review. However, it offers a glimpse into the current literature. A systematic review on the topic would paint a fuller picture of current trends in terms of access, availability and quality of perinatal health care for migrant women, a gap that should be addressed in future research.

## CONCLUSIONS

Assessment of the prevalence of and risk factors for antenatal mental disorders in migrant and refugee pregnant women is most important for managing healthcare services. There is also a profound need for healthcare professionals to be able to assess the risk factors of antenatal depression in all migrant and refugee pregnant women. Perinatal healthcare professionals should be encouraged to be culturally sensitive and to achieve cultural competency, including training in modalities such as compassion, so as to facilitate access to healthcare services and to better help women to cope with them, develop trusting relationships allowing them to reveal their depressive symptoms and to ensure acceptance of and compliance with their treatment. Mental health-related interventions such as peer support groups, group meetings, mind-body interventions, and physical exercise can improve mental health status. Mostly, integration of mental health services into primary care settings appears to be the most efficient and effective manner to facilitate the early assessment and timely treatment of perinatal mental disorders, with community midwifery playing a central role in achieving better mental health outcomes for the women and their offspring.
